# Father’s Perspectives on Family Relationships and Mental Health Treatment Participation in the Context of Maternal Postpartum Depression

**DOI:** 10.3389/fpsyg.2021.705655

**Published:** 2021-09-29

**Authors:** Cynthia L. Battle, Amalia Londono Tobon, Margaret Howard, Ivan W. Miller

**Affiliations:** ^1^Warren Alpert Medical School of Brown University, Providence, RI, United States; ^2^Butler Hospital, Providence, RI, United States; ^3^Women and Infants Hospital of Rhode Island, Providence, RI, United States

**Keywords:** fathers, partners, postpartum depression, perinatal mental health, family treatment

## Abstract

**Objective:** To understand the perspectives of fathers whose partners experienced postpartum depression, particularly (1) views on how fathers and family relationships were impacted by maternal PPD, and (2) attitudes regarding inclusion of fathers within the treatment process.

**Methods:** We conducted qualitative interviews with 8 postpartum couples using a semi-structured protocol, and administered questionnaires assessing demographics, depression, and family functioning. We abstracted data from hospital records regarding the mother’s depressive episode. We summarized quantitative data using descriptive statistics, and analyzed interview transcripts using qualitative analysis techniques, focusing specifically on fathers’ input on postpartum relationships and treatment involvement.

**Results:** Over one-third of fathers had elevated symptoms of depression, and family functioning scores suggested that most couples were experiencing dysfunction in their relationships. Qualitative analysis identified three major categories of themes, and subthemes in each category. Major themes included: (1) fathers’ experiences during the postpartum period, including not understanding postpartum mental health conditions and desiring more information, experiencing a range of emotions, and difficulty of balancing work with family; (2) fathers’ views on postpartum relationships, such as communication problems, empathy for partner, and relationship issues with other family members; (3) fathers’ attitudes toward postpartum treatment, including openness to be involved, perceived benefits, and barriers and facilitators to the inclusion of partners in treatment.

**Conclusion:** Though barriers exist, many fathers are motivated to be included in the treatment process. In addition to supporting maternal wellbeing, fathers view treatment as a means to improve issues in the couple or family system, such as communication difficulties.

## Introduction

The past decade has seen a steady increase in studies focused on postpartum fathers and partners (Darwin et al., 2021). Although most clinical, policy, and research attention remains focused on maternal mental health, there is growing recognition that many fathers and other non-birthing parents and partners face significant challenges during the perinatal period, with up to 10% suffering clinical symptoms of depression or anxiety in the months after a new baby is born (Paulson and Bazemore, 2010; Cameron et al., 2016; Leach et al., 2016). This work has helped to propel new public health and advocacy initiatives targeting the wellbeing of fathers and partners, across a range of national and international advocacy and healthcare organizations. However, much remains to be understood about fathers’ and partners’ experiences and interests in engaging in treatment.

In cases where the mother is experiencing major depression or other significant distress during pregnancy or after birth of the baby, the partner’s role becomes even more central. Partners may be the first to notice changes in a mother’s functioning or mood, and can play a critical role in accessing appropriate care. In addition, stress in the couple’s relationship can put a mother at risk for greater symptoms or a prolonged course of depression. Indeed, there are well-documented associations between relationship factors (partner support, family conflict, and relationship satisfaction) with onset and course of maternal depression during the perinatal period (Dennis and Ross, 2006; Figueiredo et al., 2018); Interconnections have been shown to exist between maternal and paternal symptomatology (Cameron et al., 2016; Figueiredo et al., 2018) such that if one parent has elevated symptoms, the other parent is more likely to have symptoms themselves. The unique vulnerabilities of each parent, including their clinical history and their individual capacity to adapt to stressful circumstances, are likely to play an important role in predicting the long-term stability of the relationship. Indeed, in line with their vulnerability-stress-adaptation model (Karney and Bradbury, 1995), the transition to parenthood can be viewed as a critical period when each individual parent’s vulnerabilities can have significant impacts on the family system Importantly, parental mental health issues in either the mother (Dunkel Schetter and Tanner, 2012) or father (Paulson and Bazemore, 2010) can have serious adverse effects on the child’s long-term development.

Because of the salience of relationship factors in perinatal mental health, partner-inclusive treatment approaches have been proposed (Milgrom et al., 2005; Cohen and Schiller, 2017). Misri et al. (2000) conducted one of the first partner-inclusive trials for postpartum depression (PPD); however, since that time, relatively few couples or family interventions have been rigorously tested. Several investigators have tested strategies for including partners in preventative or treatment interventions for PPD, yet limitations exist to this body of work (Alves et al., 2018). Although several partner-inclusive interventions have been examined for pregnant women, primarily from a psychoeducational framework (Park et al., 2020), few trials have tested partner-or family-based interventions for PPD. Some exceptions include Brandon et al. (2012) pilot study of partner-assisted interpersonal psychotherapy, and involvement of partners in Milgrom et al. (2016) internet-based PPD intervention. Because of the strong rationale to include partners and families, and paucity of work in this area, this remains an important area for future study.

As PPD interventions are developed with partners in mind, it is critical to more fully understand their experiences. Direct input from partners of women with PPD can clarify attitudes and opinions regarding inclusion in treatment, areas of desired treatment focus, and barriers and facilitators to engagement. Studies documenting postpartum fathers’ experiences have yielded important themes such as desire for education regarding perinatal mental health, feelings of helplessness, and a sense of exclusion from the treatment process. Although these studies have provided helpful information, limitations exist (Lever Taylor et al., 2018) including retrospective recall of postpartum experiences from over 10 years previously, which could be subject to recall bias, and lack of racial/ethnic diversity in the sample. Additionally, most studies only assessed the father, with little detail regarding the nature of the mother’s diagnosis, symptoms, or treatment.

In light of the critical role of partners and family members, and barriers to engaging family members in postpartum mental health care, we sought to further elucidate fathers’ experiences in the context of maternal PPD, with a particular focus on their perspectives regarding issues that arose within the family system. Given the profound impact that depression can have on maternal functioning during the perinatal period, it is important to understand fathers’ postpartum experiences in this context – including their understanding of the mother’s symptoms, how their role in the family may have changed during the episode of PPD, and their reactions to the treatment process. In addition, we sought to understand fathers’ views regarding being more directly involved in PPD treatment – including potential benefits, barriers, and strategies to promote participation.

## Methods

### Participants

This study included postpartum couples in which the mother experienced a major depressive episode after the baby’s birth. Inclusion criteria included: (1) age 18 or older; (2) English speaking; (3) mother received treatment for depression within the first postpartum year at a local mother-baby partial hospitalization program. Exclusion criteria included acute distress at the time of interview (i.e., psychosis, substance use, thoughts of harming self or others). We note that quantitative data are included from both members of the couple to provide context; however, in light of the focus on fathers, thematic analysis of qualitative data focused specifically on father’s input during the interview.

### Procedures

Procedures were approved by the hospital institutional review board; all staff members were trained in the protection of human subjects and study-specific procedures.

Participants were recruited from a perinatal psychiatric partial hospitalization program (Howard et al., 2006; Battle and Howard, 2014). Once staff determined that a patient met inclusion criteria, research staff explained the study, asking screening questions, and assessing interest. If the woman and her partner were interested in participating, they attended an in-person interview. Following informed consent, they completed demographic and self-report symptom measures and took part in an audio-recorded interview. Of note, a standard component of care at the recruitment site involves staff inviting a woman’s partner or other family member to take part in a family meeting during her treatment; as such, many fathers had the experience of attending a family meeting with their partner’s provider at the treatment site.

### Assessments

#### Depression Symptoms

Postpartum women completed the Edinburgh Postnatal Depression Scale; EPDS (Cox et al., 1987), a widely used, validated 10-item measure that screens for depressive symptoms during the perinatal period; scores range 0–30, with scores 13 and higher suggesting presence of depression, and scores 19 and above considered in the severe range. Partners of postpartum women completed the Beck Depression Inventory (BDI) (Beck et al., 1988), a validated 21-item measure of depressive symptoms used with clinical and non-clinical populations. BDI scores range 0–29; scores over 10 indicate presence of depression and scores 19 and higher are considered severe. Mean scores were calculated on the EPDS and BDI; higher scores indicate higher levels of depressive symptoms.

#### Family Functioning

Participants completed the Family Assessment Device – general functioning scale (FAD-gf)(Epstein et al., 1983), a validated 12-item measure that assesses individual’s perceptions of key areas of family functioning. It has been used in perinatal samples (Blom et al., 2010; Henrichs et al., 2011). Respondents indicate the extent to which statements describe their family on a scale of 1 to 4 (e.g., “*In times of stress we turn to each other for support*”). Mean scores were computed; higher scores reflect more problems, with means over 2 suggestive of dysfunction in the family system.

#### Clinical Record Review

Women granted permission to obtain information regarding their treatment, including psychiatric diagnosis, clinical features during depressive episode, and intake depression severity on the EPDS (Cox et al., 1987).

#### Qualitative Interview

Each couple took part in a joint, 60-min audio-recorded interview with the first author following a semi-structured protocol that included open-ended questions and follow-up prompts. Interviews covered: (1) brief history of their relationship; (2) description of experiences in the family since the baby’s birth, including when the mother was depressed; (3) discussion of issues or challenges in family relationships since the baby’s birth; (4) attitudes toward mental health treatment, including the inclusion of fathers and other family members in PPD treatment.

### Data Analysis

We used SPSS version 22 to analyze the quantitative data, summarizing demographics and computing ranges, means, and standard deviations of symptom measures. Interviews were audio-recorded, and verbatim transcripts generated, with all references to participant identifiers removed. Transcripts were reviewed by the transcriptionist and lead interviewer to check for errors or inconsistencies. Participant responses to the open-ended questions were analyzed using thematic analysis (Braun and Clarke, 2006). First, one author (CB) read all transcribed interviews and developed a preliminary codebook reflecting themes that arose during the interviews; themes were organized into major topic areas based upon sections of the interview. In light of the focus on father’s perspectives, the codebook and thematic analysis process specifically captured themes arising from the father’s input during the interviews. Once the codebook was developed, the first and second author used the codebook to independently assign themes to a subset of interviews. Through in-depth conversation regarding themes applied to this body of interviews, revisions were made to the codebook, including addition of new themes or modification of existing themes, as needed. When finalized, all transcripts were coded with the final codebook. To ensure consistent application of codes, 50% of interviews (180 pages of transcripts) were coded by more than one coder, and meetings were held to review codes applied to each passage in the transcript. Although the majority of codes matched, any discrepancies were resolved via consensus. Ultimately, after all transcripts were coded, the final list of themes, and representative quotes was developed by the first and second author, and agreed upon by consensus.

## Results

### Participant Characteristics

Table 1 displays participant characteristics, including demographics and clinical information. All mothers met criteria for major depressive disorder with most exhibiting significant levels of symptoms: more than half expressed suicidal ideation, and one-third were hospitalized as an inpatient during their recent depressive episode. Three-quarters of women had EPDS scores above the clinical cut-off for depression at the time of their partial hospital admission, with 37.5% scoring in the severe range on the EPDS. The mothers’ partial hospitalization took place between 5 and 10 months postpartum (Mean = 5.5, SD = 3.9). Interviews took place between 1 and 5 months average after that admission (Mean = 2.5, SD = 1.4). At the time of the interview, maternal depression was still elevated, but generally in the mild-moderate range and not related to time since the partial hospitalization. Paternal depression, on average, was in the minimal range at the time of the interview; however, notably, over a third (37.5%) of fathers endorsing elevated symptoms – with one depressed father (12.5%) exhibiting symptoms of severe depression. In terms of family functioning, FAD-gf scores suggested dysfunction in the family system, with the mean scores for both partners in the clinical range.

**TABLE 1:** Participant demographic and clinical characteristics.

	**Fathers**		**Mothers**	
**Variable**	***n* or mean**	**% or *SD***	***n* or mean**	**% or *SD***
Age	*29.63*	*(4.43)*	*24.88*	*(5.54)*
Marital status				
Married/Cohabitating	7	87.5%	7	87.5%
Single/Not cohabitating	1	12.5%	1	12.5%
Race/Ethnicity				
White or Caucasian	2	25.0%	3	37.5%
Black or African American	4	50.0%	2	25.0%
Latino or Hispanic	2	25.0%	2	25.0%
Multiple	0	0.0%	1	12.5%
Education				
High school or Less	2	25.0%	4	50.0%
Some college	5	62.5%	4	50.0%
Completed college or graduate degree	1	12.5%	0	0.0%
Household income				
10,000–$24,000	2	25.0%	2	25.0
25,000–$49,999	1	12.5%	1	12.5
50,000–74,999	5	62.5%	5	62.5
Depression severity at time of interview				
BDI (Fathers)	7.88	10.79	–	–
EPDS (Mothers)	–	–	11.75	7.06
Family functioning				
FAD-general functioning scale	2.04	0.51	2.19	0.76
Maternal clinical characteristics during episode				
Depression severity at intake (EPDS)			17.38	7.09
Primary diagnosis of MDD			8	100.0%
MDD, single episode			4	50.0%
MDD, recurrent			4	50.0%
Psychotic features this episode			2	25.0%
Anxiety features this episode			3	37.5%
Depression onset				
During pregnancy			2	25.0%
During postpartum			6	75.0%
Suicidal ideation or plan this episode			5	62.5%
Substance abuse history noted at intake			2	25.0%
Inpatient stay during this episode			3	37.5%

### Qualitative Themes

As shown in Table 2, we organized the themes into 3 broad categories, including: (1) *Fathers’ experiences during the postpartum period* – including thoughts and feelings as individuals, fathers, and partners in the context of maternal PPD; (2) *Fathers’ views on postpartum relationships* – including relationship with his partner, others family members and the new baby; and (3) *Fathers’ attitudes toward mental health treatment* – including perspectives regarding inclusion of family members in the treatment. Below we describe major areas and sub-themes that emerged within each category.

**TABLE 2:** Qualitative themes and representative quotes.

**Topic area 1: Fathers’ experiences during the postpartum period**	
**Theme**	**Sample quotes**
Lack of knowledge of post-partum mental health conditions	“I wasn’t thinking it was a depression at the moment, I thought that she was just having a hard time” “I am sure most guys don’t know what post-partum is” “I never seen nothing like this in my life. I’ve never seen this. I’ve never even heard about it”
Empathy and desire to help partner	“Anything to help her out” “I don’t, I don’t like seeing her hurt like that” “I do want to provide the support that she need for her to get better”
Challenges of work-life balance	“I was working a lot so I wasn’t exactly home too much” “I was like extremely exhausted between helping her get through the depression, me working sort a two jobs at once, and taking care of the baby with no sleep”
Fathers’ emotional experiences and distress during the perinatal period	“I was really worried” “I was scared” “I think that mentally I wasn’t ready for all these things” “I kind of felt like I was trapped in a way” “My life seemed like it was turned upside-down” “Talking about your feelings isn’t something that normally think of as a me, or a “guy” thing to do” “I got to be strong for all of us”

**Topic area 2: Fathers’ views on postpartum relationships**	

**Theme**	**Sample quotes**

Communication problems and tension in the couple’s relationship	“The thing is that we have communication problems, we don’t talk about the problems ourselves” “The main problem we have is communication, we don’t communicate too well”
Role transitions during perinatal period and problem solving as a couple	“Talk about the ultimate test: having a baby” “I had to learn real quick [to do child care]” “Once the baby came along… we pretty much rolled uphill from there”
Feelings of love, closeness, appreciating time together	“I’d love to spend time with you like take a weekend and just go” “Bring ourselves back to the level of calmness and loving each other again” “I certainly feel like if we can get through (this) we can get through anything”
Experiencing support from and tension with family members	“If it wasn’t for my mother, I think she could have gotten over the postpartum depression [sooner]” “I do really feel stuck in the middle [of mother and wife]” “Guys at work were a big help, my parents, her parents, her family”
Relationship with baby	“I want… to make [our relationship] work because I want to be with my kid and I want to see them grow and everything” “I focused a lot on [my baby] and making sure he was taken care of” “I don’t want our kids subjected to that kind of stuff [tension with family”

**Topic area 3: Fathers’ attitudes toward mental health treatment**	

**Theme**	**Sample quotes**

Openness and benefits to being involved in treatment	“It was really great that [clinician] did actually call me if she hadn’t called me. I wouldn’t have known [what their partner was struggling with]” “It’s like I understand where she’s coming from, and it’s like I’m trying just To help her get through it” “The lady there just telling me about everything that, like telling about how she’s feeling that’s when I really understood and like it motivated me to help her”
Barriers, challenges and strategies for involving family members in treatment	“It’s hard to go up there and talk to somebody” “I think the flexibility [in scheduling treatment] would be more of an issue” “[If the therapist] came to the home? That would be awesome” “Well, if she needs you there for your support, just be there, cause you want her to do the same for you” “If you really care about the kid, you will be there [in treatment]”
	

#### Thematic Category 1: Fathers’ Experiences During the Postpartum Period

##### Lack of knowledge of postpartum mental health conditions

Most fathers (6/8) reported lack of knowledge of maternal postpartum mental health conditions. They reported either not knowing their partner was having difficulties or believing their partners were having typical issues that were expected of perinatal period *(“wasn’t thinking it was depression at the moment, I thought that she was just having a hard time”).* As summarized by one father, *“I am sure most guys don’t know what postpartum is.”* Many fathers endorsed a wish to know how to recognize mental health conditions earlier, and more knowledge about strategies to support their partner.

##### Empathy for partner

Fathers reported feeling worried, scared, frustrated, overwhelmed, or helpless by their partners’ emotional difficulties (*“I was really worried” “I was scared”*). Most fathers (6/8) voiced empathy for their partners, and a desire to help them get through this difficult time (*“I do want to provide the support that she needs for her to get better;” “I don’t want to see her like this;” “Anything to help her out”*). They also provided emotional and practical support for their partners including taking their partners to treatment appointments, supporting partners’ self-care including engaging in healthy nutrition and sleep. Some reported having to do most of the childcare while their partners were depressed.

##### Challenges of work-life balance

One of the difficulties many fathers (5/8) described was balancing work and family life during the perinatal period, a challenge that was exacerbated during the period of time when mothers were depressed. Some expressed difficulty attending as many of their partners’ prenatal or postnatal appointments as they had wished. Some noted that their need to maintain employment made it hard to notice that their partners were struggling emotionally. Many fathers discussed a desire to take time off of work during the period when their partners were depressed, yet for some it was not possible. Fathers who maintained regular working hours when their partner was depressed reported feelings of exhaustion from the joint responsibility of going to work full time, and also caring for their baby and partner.

##### Fathers’ emotional experiences and distress during the perinatal period

All fathers (8/8) brought up the range of emotions, and often the distress, that they experienced during the postpartum period. Many fathers expressed the feeling that they were not prepared for the perinatal period (“*I think that mentally I wasn’t ready*” “*The first week home for me [with the baby] – ‘Oh my God’*”). In addition, many noted challenges were compounded and intensified by their partner having a mental health condition. They describe this period as “*surreal*” and “*my life felt like it was turned upside down*.” They reported feeling sleep deprived, run-down, and tired. Some expressed missing their pre-parenthood life when they were able to enjoy greater sleep and more time with friends.

Fathers indicated that they felt scared about their partners’ health and the wellbeing of the baby. They were concerned about when or if their partners were going to get better, and whether they would be able to manage caring for both the baby and their partners. A few fathers expressed anger, feeling their experience was unfair, missing out on the postpartum experience they hoped for (“*Everybody knew I was angry it’s unfair – she’s missing out on him, and I am missing out of both of them. We couldn’t be a family*”). Other fathers expressed regret and guilt that they did not seek help earlier for their partners, or did not recognize symptoms sooner (“*I should have taken her to the doctor then*,” “*I really didn’t know. I know she was having a hard time with the baby but never thought she was having those kinds of thoughts*”).

Although fathers described a wide range of emotions that they experienced during the postpartum period, many reported a sense that it was not acceptable to have these feelings, or that they were unsure how to express them. (*My mood didn’t matter what I felt was not a concern,” “I couldn’t get depressed, that wasn’t, that couldn’t not an option”*). Some made a connection between limited expression of emotion and their gender (“*Talking about your feelings isn’t something that normally think of as a me, or a guy thing to do*”).

#### Thematic Category 2: Fathers’ Views on Postpartum Relationships

A number of themes emerged regarding postpartum relationships, including the couple’s relationship, relationship with the new baby and with other family members such as in-laws and siblings.

##### Communication problems and tension in the couple’s relationship

All fathers (8/8) reported either some form of communication problems, or an increase in tension during the perinatal period, often relating to managing care for the baby and adjusting to parenthood. Tension sometimes stemmed from differing communication styles and problems (*“The thing is that we have communication problems. We don’t talk about the problems”*). Fathers often believed that tension and communication problems were compounded by their partners’ depressive symptoms- in particular, increases in partners’ irritability. In some cases, fathers reported that they did not want to hurt partners’ feelings by sharing concerns. Therefore, many fathers reported that during arguments they tried to stay calm and not argue back, which in turn decreased communication. Some mentioned their partner did not communicate feelings directly, making it difficult to understand her level of distress. A few believed that if they had better communication, this may have prevented a progression of their partner’s depression.

##### Role transitions during the perinatal period and problem solving as a couple

All fathers (8/8) described role transitions the couple underwent during the perinatal period including establishing co-parenting routines and finding new ways of relating to each other. Some found adjusting to co-parenting routines easy, while others did not. Many felt that the communication as a couple changed during this period, and for those with prior conflict, disagreements intensified with the demands of the postpartum period. Some fathers felt that their partners changed and were not the same person they were prior to pregnancy. To cope, some couples developed new ways of problem solving and communicating. For example, one couple developed a strategy of going out to eat in a public space, to be “forced” to calmly talk about issues (“*we have to be civil when we are out in public*”).

##### Feelings of love, closeness, and appreciating time together

Many couples (6/8) reported not having sufficient time or opportunity to be together as a couple (“*I’d love to spend time with you – like, take a weekend and just go*”). Some fathers noted that postpartum experiences had made them feel closer and stronger as a couple, while others reported the opposite.

##### Experiencing support from and tension with family members

Many fathers (6/8) reported receiving practical and emotional support during the postpartum period from their in-laws, parents, siblings, extended family, and friends. Most support was related to childcare, but some fathers reported receiving emotional support. Conversely, many fathers (6/8) described varying degrees of tension with family members, especially the couple’s parents (grandparents of the baby). Many fathers described differences in opinion related to childcare; some reported that grandparents could be critical of their partner. This led many fathers to feel “*in the middle.*” Some reported that the tension with their family worsened the distress of the couple and of their partner (“*[tension with the mother-in-law] made the depression worse*”).

##### Relationship with baby

Fathers reported the birth of the baby as both a source of stress, and a motivation for change and growth. Many fathers (4/8) reported infant difficulties with sleeping, feeding, and crying as sources of stress. However, they also reported that their babies were a motivation for learning how to be a father, improving their relationship with their partner, and supporting their partner through their depression (“*we can’t be like this it’s not healthy for the two children*” *“I had to be there for the baby”).*

#### Thematic Category 3: Fathers’ Attitudes Toward Mental Health Treatment

##### Openness and benefits to being involved in treatment

As noted earlier, most fathers participated in a family meeting during their partner’s treatment. Fathers universally reported this as helpful in promoting understanding of PPD, and how to support her (“*The lady there just telling me about everything that, like, telling her about how she feels that’s when I really understood it motivated me to help her*”). Some felt that the family meeting was helpful in improving the couple’s communication, including having someone mediate the conversation when there was tension. When asked about important areas of focus for postpartum family or couples treatment, many endorsed communication, problem solving strategies, and promoting increased time together as important goals. They also emphasized the importance of PPD education, “warning signs” and strategies to address PPD with their partner. In all, the vast majority of fathers interviewed (7/8) remarked that they were open to engaging in family treatment during the postpartum period and viewed it to be potentially helpful.

##### Barriers, challenges, and strategies for involving family members in treatment

Many fathers (6/8) identified barriers or challenges with becoming involved in their partner’s PPD treatment. Specifically, some fathers expressed finding the family meeting helpful and desired more, while others found it time-consuming and disruptive to work. There was a reported preference for evening or weekend appointments and childcare assistance for older children. Some preferred to have sessions at locations that were familiar, including the obstetrical hospital where they received prenatal and postnatal care are even in their homes. Some fathers suggested a “fathers’ only” peer treatment group could be beneficial; however, other fathers reported feeling uncomfortable sharing information in groups, and preferred privacy, e.g., participating in online forums to access resources on postpartum mental health conditions.

## Discussion

Fathers and partners are largely left out of perinatal research and clinical care, in spite of their impact on the wellbeing of mothers and infants during the postpartum period. The current findings shed light on the experiences of fathers who have a partner struggling with significant symptoms of depression, representing families who are more at risk for adverse outcomes and arguably in most need of services. Not surprisingly, in addition to the high levels of depression among the mothers, many fathers exhibited depression symptoms themselves, and family functioning scores in the clinical range. Our analyses found that fathers reported multiple challenges when their partner was experiencing depression symptoms, including feeling exhausted, unprepared, uninformed regarding maternal postpartum illness, and experiencing difficulty balancing work and family needs. Second, fathers were universally open to engaging in various forms of psychosocial and mental health treatments, although many described logistic and personal barriers. Overall, these results add to the growing evidence of the importance of including fathers and partners in perinatal research and clinical care in the context of maternal PPD.

Consistent with prior studies regarding concordance of parental depression, many partners of mothers with PPD reported elevated depression; however, regardless of depression status, all fathers endorsed strong emotional experiences during the postpartum period. Fathers expressed a wide range of emotions –fear, guilt, anger, worry, and exhaustion, echoing other research with postpartum fathers (O’Brien et al., 2019). In spite of fathers voicing an array of strong emotions during the research interview, many also noted that they had trouble expressing their feelings, or felt they needed to prioritize their partners’ needs. Prior research has similarly found that postpartum fathers may question the legitimacy of their feelings and needs, particularly when they have a depressed partner (Darwin et al., 2021). Our sample was diverse in terms of race and ethnicity and socio-economic background; though not directly examined in the current study, it is possible that cultural norms and beliefs could impact father’s views regarding expression of emotions.

Fathers voiced feelings of empathy and a desire to help with partners, but also acknowledged problems in the relationship, such as communication issues, impaired problem-solving, and difficulty managing parenting tasks. This distress was also reflected in the self-report family functioning scores which on average reached clinical levels. Thus – couples were struggling with serious issues, some apparently long-standing, and other issues were exacerbated with the transition to parenthood and onset of maternal depression. The fact that all mothers and many fathers in the sample struggled with symptoms of depression may have shaped their assessment of their relationship, potentially leading to a more negative appraisal in some cases.

Although relationship challenges were universal in our sample, similar to other samples of postpartum men (Marrs et al., 2014), we also found that fathers endorsed openness to being involved in mental health treatment. They noted that treatment could help improve problems in the relationship and help them communicate and find ways of spending more time together. Additionally, consistent with prior research (Letourneau et al., 2011; Mayers et al., 2020) fathers were eager for information regarding PPD and wanted to learn about specific ways they could provide support for their depressed partner. However, many wished they had received information sooner, to help identify problems, potentially preventing progression of the mother’s illness and the impact on the family system. There was a generally positive acceptance of mental health treatment, but fathers noted that personal and logistic barriers impeded attendance. To fathers, the new baby and other children represented strong motivators for engaging in treatment as did supporting their partner’s recovery. Fathers also voiced interest in flexible scheduling, location, and onsite childcare, as well as interest in various modalities (couples/family treatment, peer/partner groups, and online forums). Although the motivation for treatment among postpartum couples may stem from the need to address PPD or other maternal symptoms or functioning issues, treatment approaches that provide support specifically for fathers and partners to manage their own distress – and to discuss their vantage point regarding role changes and challenges – are critically important, including sensitivity to differing levels of comfort that partners may have with emotional expression. For many families, engaging in professional mental health treatment may be new; efforts to make the treatment experience accessible, pleasant, and directly useful for fathers and partners will likely help promote greater engagement.

Building on these findings, future maternal mental health interventions should consider expanded strategies to include partners in the treatment process. It will be important for services to be designed in such a way that family involvement is feasible, with a flexible format, scheduling, welcoming the presence of the baby, and potentially offering home-based services. Additionally, building on existing internet-based interventions for perinatal women (O’Mahen et al., 2014; Milgrom et al., 2016), it will be important to develop or expand digital health interventions that more centrally involve partners and other family members.

Strengths of the present study include assessment of both paternal and maternal depression and including data from the clinical record regarding the mother’s depressive episode to contextualize the couples’ postpartum experiences. In addition, our sample, though small, had diverse representation across a range of racial and ethnic groups and socioeconomic backgrounds, consistent with the population in our geographic region in the northeastern United States. In terms of limitations, our sample size is small and in these analyses we specifically focused on the paternal experience. Given that some participants were remarking upon a more recent postpartum experience and treatment episode (e.g., past 3 months), and others were remarking on an experience that was earlier (e.g., 6–9 months ago), there may be variability with regard to how clearly events are recalled. Although the sample is diverse in terms of racial and ethnic background, it may not reflect the views of all postpartum fathers. We did not have any same-sex couples or gender diverse individuals. Understanding the experiences and treatment needs and preferences of diverse families and families with non-traditional structures will be important in future research. In addition, other family structures should be considered in developing new treatment approaches that explicitly include other support persons particularly for single parents who may not live with a partner or co-parent (Alves et al., 2018). Accurate screening and identification of mental health concerns in *both* parents and effective treatments that promote active involvement of key family members and support persons, will allow for more effective reduction of stress during the perinatal period, and ultimately help prevent long term adverse outcomes for children and families.

## Data Availability Statement

The data that support the findings of this study are available from the corresponding author upon reasonable request.

## Ethics Statement

The studies involving human participants were reviewed and approved by Butler Hospital Institutional Review Board. The patients/participants provided their written informed consent to participate in this study.

## Author Contributions

CB conceptualized the study, conducted quantitative and qualitative data analyses, and prepared the manuscript. ALT contributed to the qualitative data analysis and prepared portions of the manuscript. MH and IM helped in the initial planning stage of the study and provided editorial suggestions for the final manuscript. All authors contributed to the article and approved the submitted version.

## Conflict of Interest

The authors declare that the research was conducted in the absence of any commercial or financial relationships that could be construed as a potential conflict of interest.

## Publisher’s Note

All claims expressed in this article are solely those of the authors and do not necessarily represent those of their affiliated organizations, or those of the publisher, the editors and the reviewers. Any product that may be evaluated in this article, or claim that may be made by its manufacturer, is not guaranteed or endorsed by the publisher.
